# Factors influencing wildlife roadkill in the Ngorongoro Conservation Area, Northern, Tanzania

**DOI:** 10.1371/journal.pone.0323994

**Published:** 2025-05-14

**Authors:** Gregory A. Mtega, Victoria Shayo, Richard D. Lyamuya, Gabriel Mayengo

**Affiliations:** 1 Department of Ecological Monitoring, Ngorongoro Conservation Area Authority, Karatu, Tanzania; 2 Tanzania Wildlife Research Institute (TAWIRI), Arusha, Tanzania; 3 Department of Wildlife Management, College of African Wildlife Management, Moshi, Tanzania; Universidade Federal de Mato Grosso do Sul, BRAZIL

## Abstract

Globally, extension of road network is among the major threats affecting different fauna survival. Roads traversing protected areas, usually harm wildlife species (e.g., wildlife roadkill). Wildlife roadkill is of global conservation concern and has been reported to occur in different protected areas worldwide. Very little information on the problem is currently available in the Ngorongoro Conservation Area (NCA). The purpose of this study was to identify factors that affect wildlife roadkill in the NCA. The 82-kilometer main road from Lodoare to Golini served as the study’s transects. Data was collected for a duration of one year from July 2021 to June 2022. Vehicle moving with a speed limit of 20km/hr were used during the data collection. The survey started early in the morning from 07:00 am to 06:00 pm and employed both direct and opportunistic encounter observations for recording wildlife roadkill incidences. The results revealed that 85 individual animals belonging to 21 families, i.e., 5 mammalian, 3 reptilian, and 10 bird orders were recorded killed within one year period in the area. These animals comprised 26 different species, which included 10 mammalian, 5 reptilian, and 11 bird species. Moreover, more birds (69.4%) than mammals (18.8%) and reptiles (11.8%) were found killed in the area. Additionally, the night jars (*Caprimulgus europaeus*; 30.6%) followed by black rat (*Rattus rattus*; 7.1%) and chameleon (*Chamaeleo chamaeleon*; 3.5%) were the most frequently recorded killed species in the area. Furthermore, wildlife roadkills did not differ significantly between seasons (p = 0.371) and time of day (e.g., morning vs. afternoon; p = 0.652) but differed significantly between their body size (e.g., small, medium, and large; p < 0.001) and habitat types (e.g., grassland, woodland, shrubland, wooded grassland, forest; p = 0.005). The study recommends punishments and penalties for overspeeding drivers and installing cameras, speed limits, and signboards along the highway to alert drivers to reduce speed. Further, providing regular education to road users on the impacts of roadkill within the ecosystem is critical.

## Introduction

Roads that cross protected areas are built to simplify movement of goods and services, but they have been shown to negatively affect wildlife resources in various protected areas worldwide due to roadkill incidents [1–3]. These roads are expected to grow in size, number, and traffic in years ten to come [1,3] and some might be upgraded from unpaved into paved roads shortly including the NCA.

Worldwide, there are about 36 million km of road networks [4]. It is estimated that an additional 25 million km of roads by 2050 [4,5]. Expansion of road network is also expected in developing countries containing exceptional biological diversity and majority of hotspot areas [6]. In India, roadkill incidences were reported to highly threaten lives of many native wild animals and birds [7]. It is estimated that 340 million birds killed on the roads annually in the USA and 13.8 million birds annually in Canada [3]. In a 13 years monitoring that was done in Changbai Mountains of northeast China, a total of 13, 309 vertebrate roadkill belonging to 91 species with a roadkill rate of 6.35 ± 2.41 per 100 km per day was recorded [8]. In sub-Saharan Africa, roads in protected areas were found to highly affect animals foraging site, habitat, and acting as barriers to access corridors for their movement [9]. Due to forecasted impacts on biodiversity due to expansion of road networks worldwide, evaluating their current and future impacts is critical for improving current management practices and biodiversity conservation [1,10].

The NCA which is a UNESCO World Heritage Site and part of the Serengeti-Ngorongoro Man and Biosphere Reserve [11] is not an exceptional to this scenario. Since, in the course of supporting tourism and administration activities, anti-poaching operations, and community development, the Ngorongoro Conservation Area Authority (NCAA) has established over 1300 km long road networks classified based on various categories of road use [11]. The established road networks connect different tourist attractions, and villages as well as link the Northern Tanzania circuit Arusha, Manyara, Kilimanjaro, and Tanga with the lake zone regions (Mara, Shinyanga, Simiyu, and Mwanza) [12]. The main road from Lodoare to Golini (82 km) bisects the conservation area, allowing passage of vehicles carrying services such as goods, food, and people [11].

Moreover, between 1997 and 2013, vehicles entering the NCA increased from 24,164 to 129,968 [13,14]. With the expected increase in tourist demands in line with vehicle traffic, NCAA has planned to construct and upgrade different roads to intensify the accessibility of natural attractions as a strategy to diversify tourism activities. Yet the increased road network and upgrading of roads is a major concern in wildlife conservation and priority research in road ecology due to its potential impacts on wildlife and the ecosystem health at large [10,14–16]. In addition, previous studies have found that linear infrastructures such as roads have negative impacts including habitat loss and fragmentation, disturbance due to edge effects, and wildlife-vehicle collisions leading to road mortality [15–18]. Most roadkill studies are conducted in developed countries, but there are limited information on the impacts of roads on wildlife in the African countries [19]. Only partial information exists on wildlife roadkill’s within the road networks of NCA [1].

Therefore, this study aimed at determining factors influencing wildlife roadkill in the area. The study firstly hypothesized that small bodied animals would be more hit by vehicles compared to medium to large animals along the Lodoare- Golini Road in NCA. Secondly, the frequency of wildlife roadkill would occur differently between habitat types within the NCA. The information accrued from this study would help in minimizing the impacts of wildlife roadkills and increase the safety of people and their properties within NCA. Further, it will create awareness and issues to consider for different stakeholders in designing and expanding road networks, especially within protected areas.

## Materials and methods

### Study area

The study was conducted in the Ngorongoro Conservation Area (NCA), which covers 8,292 km^2^ [20]; NCA is in the North of Tanzania 90 kilometers West of Arusha, neighbouring South-eastern edge of Serengeti National Park between 2°30′ to 3°30′S and 34°50′ to 35°55′E [13]. NCA was inscribed on the World Heritage List in 1979, hence a UNESCO World Heritage site. The main feature of the NCA is the volcanic Ngorongoro Crater, the largest unbroken caldera in the world, home to the densest wildlife population of some 25,000 large animals, mainly ungulates alongside the highest density of diverse mammalian predators in Africa. The area supports Greater Wildebeest Migration from the Serengeti - Maasai Mara Ecosystem [21]. NCA is home to critically endangered Black Rhino and wild dog populations. The main wildlife species found include elephants (*Loxodonta africana*), wildebeests (*Connochaetes taurinus*), impala (*Aepyceros melampus*), eland (*Taurotragus oryx*), zebra (*Equus quagga*), black rhinoceros (*Diceros bicornis*) and hippopotamus (*Hippopotamus amphibius*), leopards (*Panthera pardus*), lions (*Panthera leo*), cheetahs (*Acinonyx jubatus*) and spotted hyenas (*Crocuta crocuta*) and variety of bird species [14,21,22]. This outstanding concentration of wildlife species constitutes one of the natural wonders of the world attracting tourists from different parts of the world. The area also covers the Empakaai and Olmoti Crater, Laetoli, and Olduvai Gorge, famous for their geological attractions and associated first human being to walk on earth studies [13]. The variations in climatic conditions, different landforms, and altitude have resulted in occurrence of several ecosystems and diverse habitats, with short grass plains acting as calving grounds of wildebeest, water-catching highland forests, scrub heath, montane long grasslands, and high open moorlands with evergreen trees and grasses. The vegetation of the area varies from Montane Forest, grasslands, Acacia woodlands, and thorn scrub [11]. The pastoralists, Maasai inhabit the NCA before the protected areas were created. The Maasai were allowed to live with wildlife after NCA was formed in 1959 as a multiple land use. The NCA is the home of biodiversity (flora and fauna) where forest cover plays a great role in maintaining the existence of wildlife. The NCA is located in the highlands with moist and misty where temperatures in the semi-arid plains can fall as low as 2°C, and often rise to 35°C [13]. The annual precipitation falls between November and April and varies from 500 mm on the arid plains in the West to 1,700 mm on the forested slopes in the East [13]. The area experiences short rains and long rains with an average annual rainfall of 600–900 mm [23,24], average annual temperatures of 9°C–22°C in the wet season, and 18°C–31°C in the dry season [24]. The main road that bisects the NCA runs from Lodoare to Golini gate, covering 82 km. the road connects northern Tanzania and Lake Zone regions [12].

### Roadkill assessment

The collection of the wildlife roadkill data started early in the morning from 07:00 am to 06:00 pm, twice a day for eight consecutive days, per month during the wet and dry seasons from July 2021 to June 2022 to document wildlife roadkill. Prior to data collection, standardized procedures for data recording were conducted to ensure data collection consistency. Roadkill assessment was done in an 82 km stretch of unpaved main road that connects NCA and Serengeti National Park (SENAPA) from Lodoare gate - Golini for transportation services to the area [12]. Two sampling periods were conducted daily: the morning period (07:00 am–11:30 am) as the forward direction and the afternoon (02:00 pm–06:00 pm) as the backward direction along the same road acted as a transect. The surveyed transect was divided into 1 km segments (n = 82 segments) for sampling and in total, there were 82 sampling segments along the 82 km transect. Each transect/road was driven twice a day (morning and afternoon) and therefore involved total segment sample sizes of 164 km for the study transect per day. In addition, the transect/road was driven four times per month, times 10 months (i.e., covering both wet and dry seasons), which resulted in a total effort of 6560 km of driven transects. The period also covered both the low and high tourism seasons to capture variations in the wildlife roadkills in both seasons as the number of vehicles entering the NCA varies according to seasons [10,25].

Moreover, a vehicle was used to conduct road surveys from Lodoare to Golini gate (transect) with a speed limit of 20 kilometers per hour for recording roadkill data. Furthermore, whenever opportunistic encounters of the wildlife roadkill outside the survey period but within the transect were observed, were also recorded [25,26]. The four observers in the car (two on the right, two on the left) conducted observations on either side of the road to locate any carcasses. At points where a carcass was sighted, the vehicle stopped. Morphological identification was used to identify each species killed, number of dead individuals, the location (GPS coordinates) where the roadkill was found, the date (i.e., start and end dates), and time of the day (i.e., morning, afternoon, evening) when it was found, habitat type (i.e., grassland, woodland, wooded grassland, shrubland, forest) of the area, were recorded on a standard data sheet. After all of the required data was collected, the carcasses were removed from the road to avoid double counting.

### Data analysis

The Statistical Package for Social Science (SPSS, version 16.0) and Palaeontological Statistics (PAST 4.03) program [27] software were used to analyze the data. The collected data were organized and cleaned using a Microsoft Excel spreadsheet, and a Shapiro-Wilk test was employed for testing normality. Since the results indicated that the data was not normally distributed, a nonparametric test was used to analyze the data. Differences in wildlife roadkills between the number of species killed, the IUCN status, seasons, and habitat types were tested using the Chi-square test. The differences between the wildlife roadkills between the morning (07:00 am to 11:59 am) and afternoon (12:00 pm to 06:00 pm) hours were assessed using a Mann-Whitney U test and between their body sizes (i.e., small = < 30 kg; medium = 31–50 kg; large = > 50 kg) were assessed using the Kruskal-Wallis test. Statistical significance was defined at p < 0.05.

## Results

### Diversity and composition of wildlife roadkill

The study results revealed that 85 individual animals (28 and 57 in the dry and wet seasons, respectively) belonging to 21 families, and 5 mammalian, 3 reptilian, and 10 bird orders were recorded killed during 10 field trips in the area. These animals comprised 26 species, which included 10 mammalian, 5 reptilian, and 11 bird species at an overall rate of 0.065 roadkill km per month. Moreover, more birds (n = 59; 69.4%) than mammals (n = 16; 18.8%) and reptiles (n = 10, 11.8%) were found killed in the area. Hence, birds are more affected group due to roadkill in the area. Amongst the bird species, the night jars (*Caprimulgus europaeus*; n = 26, 30.6%) were most frequently recorded killed followed by black rat (*Rattus rattus*; n = 6, 7.1%) on the mammals’ side and chameleon (*Chamaeleo chamaeleon*; n = 3, 3.5%) on the reptiles’ side (Table 1). An average of 164 kilometers was surveyed per day, which resulted a total effort of 131,200km of driven transects per year. In addition, the number of wildlife roadkill did not differ significantly in the area (X^2^ = 14.408, df = 26, p-value = 0.9672). Moreover, regarding conservation status, the wildlife roadkill differed significantly based on their IUCN status (X^2^ = 3.771, df = 125, p < 0.001), as one of them was found only endangered (Secretary bird, 1.2%) and the majority were least concerned (90.2%). While the remaining belonged to near-threatened (8.6%) IUCN status. Least concern species are plentiful in the wild, their chances of being affected by roadkills incidences is higher.

**Table 1:** Wild animal Species found killed along the Lodoare-Golini Road transect.

S/N	Common name	Body size	IUCN Status	Scientific name	Class	Order	Family	Number killed	Percent
Mammals									
1	African buffalo	Large	*NT*	*Syncerus caffer*	*Mammalia*	Artiodactyla	Bovidae	1	1.2
2	Bat-eared fox	Large	*LC*	*Otocyon megalotis*	*Mammalia*	Carnivora	Canidae	1	1.2
3	Black rat	Small	*LC*	*Rattus rattus*	*Mammalia*	Rodentia	Muridae	6	7.1
4	Bushbuck	Medium	*LC*	*Tragelaphus scriptus*	*Mammalia*	Artiodactyla	Bovidae	1	1.2
5	Dik-dik	Medium	*LC*	*Madoqua kirkii*	*Mammalia*	Artiodactyla	Bovidae	1	1.2
6	Common Duiker	Medium	*LC*	*Sylvicapra grimmia*	*Mammalia*	Artiodactyla	Bovidae	1	1.2
7	Olive baboon	Medium	*LC*	*Papio anubis*	*Mammalia*	Primates	Cercopithecidae	1	1.2
8	Thomson’s gazelle	Medium	*LC*	*Eudorcas thomsonii*	*Mammalia*	Artiodactyla	Bovidae	2	2.4
9	Wildcat	Small	*LC*	*Felis silvestris*	*Mammalia*	Carnivora	Felidae	1	1.2
10	Zebra	Large	*NT*	*Equus quagga*	*Mammalia*	Perissodactyla	Equidae	1	1.2
Reptiles									
11	Agama lizard	Small	*LC*	*Agama agama*	*Reptilia*	Squamata	Agamidae	2	2.4
12	Black mamba	Small	*LC*	*Dendroaspis polylepis*	*Reptilia*	Squamata	Elapidae	2	2.4
13	Chameleon	Small	*LC*	*Chamaeleo chamaeleon*	*Reptilia*	Squamata	Chamaeleonidae	3	3.5
14	Leopard tortoise	Small	*LC*	*Stigmochelys pardalis*	*Reptilia*	Testudines	Testudinidae	2	2.4
15	Puff adder	Small	*LC*	*Bitis arietans*	*Reptilia*	Squamata	Viperidae	1	1.2
Birds									
16	Ashy Starling	Small	*LC*	*Lamprotornis unicolor*	*Aves*	Passeriformes	Sturnidae	1	1.2
17	Bee-eater	Small	*LC*	*Merops apiaster*	*Aves*	Coraciiformes	Meropidae	1	1.2
18	Crested Francolin	Small	*LC*	*Ortygornis sephaena*	*Aves*	Galliformes	Phasianinae	7	8.2
19	Helmeted guineafowl	Small	*LC*	*Numida meleagris*	*Aves*	Galliformes	Numididae	9	10.6
20	Kori bustard	Small	*NT*	*Ardeotis kori*	*Aves*	Otidiformes	Otididae	2	2.4
21	European Night jar	Small	*LC*	*Caprimulgus europaeus*	*Aves*	Caprimulgiformes	Caprimulgidae	26	30.6
22	Spotted Eagle-Owl	Small	*LC*	*Bubo africanus*	*Aves*	Strigiformes	Strigidae	5	5.9
23	Pied crow	Small	*LC*	*Corvus albus*	*Aves*	Passeriformes	Corvidae	1	1.2
24	Secretary bird	Small	*EN*	*Sagittarius serpentarius*	*Aves*	Accipitriformes	Sagittariidae	1	1.2
25	Superb starling	Small	*LC*	*Lamprotornis superbus*	*Aves*	Passeriformes	Sturnidae	3	3.5
26	Weaver	Small	*LC*	*Ploceus cuculatus*	*Aves*	Passeriformes	Ploceidae	3	3.5
	**Total**							**85**	**100.**

NT, Near Threatened; LC, Least Concern; EN, Endangered.

### Spatial-temporal patterns of wildlife roadkill

In most of the wildlife, roadkill incidences occurred in the wet (n = 57, 67.1%) compared to the dry (n = 28, 32.9%), season but their differences were not statistically significant (χ^2^ = 29.831, df = 28, p = 0.371). Despite that in total, birds were mostly frequently recorded killed in both wet and dry seasons (n = 45, n = 12) compared to mammals (n = 7, n = 9) and reptiles (n = 5, n = 7) respectively in the area. Moreover, the occurrences of wildlife roadkill differed significantly between the habitat types in the area (X^2^ = 204.73, df = 156, p = 0.005) with more roadkill recorded in the woodland (n = 35, 41.2%), followed by grassland (n = 25, 30.6%), shrubland (n = 19, 22.3%), forest (n = 3, 3.5) and wooded grassland (n = 1, 2.4%). However, the spatial distribution of the road kill indicates that incidences occurs throughout the road. More wildlife roadkills were recorded in the morning (n = 53, 62.3%) especially Night jars (n = 15) on the birds’ side, black rats (n = 4) on the mammals’ side, and chameleons (n = 2) on reptiles’ side than at afternoon (n = 32, 37.7%) hours, no significant differences were observed between those hours (z = 0.63, p = 0.652). The wildlife roadkill differed significantly in their body sizes Kruskal-Wallis test (χ^2^ = 28.94, p < 0.001), with more having small-body size (n = 79, 92.9%), followed by medium-body-sized (n = 4, 4.7%) and large-body sized (n = 2, 2.4%). Small body sized are plenty in the wild than large body sized, hence higher chances of being affected by roadkill.

## Discussions

Worldwide, wildlife species have to maneuver through roads considered as modified landscapes, and face the risk of a collision with a vehicle on the road [28]. The study findings show that different taxa were involved in roadkill cases within NCA as had been previously reported to occur elsewhere in the world [1,2,25,29]. Therefore, they confer to previous findings, that wildlife taxa in most protected areas around the world, and more specifically in NCA are impacted by roadkill accidents. This study found that more road kill incidences occurred during wet season compared to dry season. This might be the result of increased animal activity during wet season [30,31], poor visibility of drivers [32,33], waterlogged habitats forcing animals to use avoided areas such as roads during wet season might attract certain species especially in arid and semi-arid areas where moisture is scarce [34]. Again, the study found that more bird species were impacted by roadkill cases compared to other taxa within NCA, similar to those reported previously [1,2,35]. A study that was done in Latin America found similar observation that birds are highly affected taxa in road kill incidences [36]. Similar findings were observed by [28] in South Africa. In a study that was done in Northern Tanzania by [25] found that birds were the most frequently killed taxon, followed by mammals, reptiles and amphibians. This could be because birds are known to search for food on roads more likely than other taxa and, hence, become more prone to roadkill [35]. Bird roadkill may occur while they are either perching on or near the road and are scared away by the oncoming vehicle, or they may clash when flying across the road at a low elevation [25,29,35]. In addition, heat stored on the road surface is released into the atmosphere at night, creating heat islands around roads, birds and snakes preferentially aggregate on or near warm roads, increasing their risk of being hit by cars [37]. Moreover, many bird species are probably put in danger in traffic because of over speeding of drivers as birds lack the cognitive ability to swiftly digest all the information about distance, direction, and speed from vehicles [35].

Amongst the birds in our study, the nocturnal Night jars (*C. europaeus*) were most frequently killed. This might be contributed by their habit of settling on roads in the open space and warmth of the road surface attracting them, particularly in the cooler evening or early morning hour [38]. Further, headlights of cars stun nightjars, hence they are considered as traffic casualties species [39]. Due to its altitude, topography and temperature inversion, NCA is often shrouded in fog, particularly in the early mornings or during the rainy season. Majority of drivers turn on headlights of their cars for increased visibility, detect road hazards and vehicle visible to others reducing the likelihood of accidents [40]. A study that was done by [2], found higher road mortality likely to occur in birds with smaller bodies, higher population densities, longer lifespans, omnivorous diets or diets predominately made up of plants and seeds. Explanation killing birds could be that the late drivers who want to get out of the NCA gate before 06:00 pm or late hours are overspeeding their vehicles while at the same time, the vehicle’s lights are on to an extent that they blind the birds’ eyes and also do not give a chance for the species to take off and run away [1,41]. The study also recorded the mortality of Secretary Birds (*S. serpentarius*), a species listed by the International Union for Conservation of Nature (IUCN) as endangered [42]. Previous studies found that Secretary Birds prefer road surfaces as hunting grounds for capturing different prey species or for taking advantage of the presence of carcasses on road, which consequently exposes them to vehicle collisions [1]. When roadkill impact secretary birds, it significantly result into their population decline, reducing breeding success and decrease their genetic diversity [43].

Furthermore, this study found that few numbers of mammals (n = 16) were recorded killed by vehicles likely because they spent very short time around the roads. After all, food and water are limited near roads [1]. Moreover, probably they might have been scavenged or removed by other animals/people in the area [44]. On the other hand, the Black rat (*Rattus rattus*) was the one species frequently hit by vehicles in the area. This might have been caused by their strong preference for feeding along vegetated road verges or sometimes due to their high population density [1,45]. Other contributing factors could be the fact that they are small in size which makes them more difficult for drivers to detect their presence and avoid those [46].

Despite that reptiles are most likely to be victims of roadkill’s due to their distinguishing long body morphology and their behaviour of frequently using roads as thermoregulation sites [47] but again, few numbers of them were recorded killed in NCA, and most representing the chameleons (*C. chamaeleon*). In a study that was done across Europe, reptile casualties was found more pronounced compared to other taxa which is in agreement with current study [48]. This could be probably because this species is a slow-moving reptile, and therefore commonly hit by vehicles [47].

Interestingly, the study recorded only two killed carnivore species including bat-eared fox (*Otocyon megalotis*), and wildcat (*Felis silvestris*). These animals were probably killed while either foraging or crossing the road or due to their inability to escape from overspeeding vehicles [1]. When water is scarce, carnivores normally expand their home ranges and often cross roads through their expanded territory [28]. This might lead them into roadkill cases. Additionally, the wildlife roadkills with small body sizes (<30 Kg) were most frequently recorded killed compared to medium body-sized (31–50 Kg) and large-body sized (>50 Kg) probably because they are small in size which makes them more difficult for drivers to see and avoid [47].

The study results again found that wildlife roadkills occurred mostly in woodland habitats in the area. This finding strongly agrees with that found in southern Portugal, where there was a strong relationship between vulnerability to traffic deaths and foraging behavior and habitat type, with the most vulnerable species being the small woodland species that prefer to feed in shrubs and trees in the area [2,49]. Furthermore, ecological and behavioral characteristics increases the vulnerability of some species [2]. In addition, in woodland habitats probably there are more forage resources, shelter, and cover for most of the wildlife found in the area, and thus the increased movements encountered during searching for those resources [50] could have increased their chances for collision with vehicles. Additionally, the type of habitat close to the roads can attract different wildlife species especially birds to the roads and increase their risk of becoming roadkill victims [47]. Furthermore, [51] reported that high species richness areas were having more roadkill incidences compared to low species richness areas.

## Conclusion and recommendations

The present study recorded a substantial number of reptiles, mammals, and birds as roadkill victims on a stretch of the 82 km main road located in the NCA. The loss of individual wildlife species by roadkill has negatively impacted the structure of the terrestrial wildlife community and caused a severe loss of biodiversity in the area. Therefore, study recommends enforcing punishments and penalties for overspeeding drivers, installation of cameras to monitor speed limit especially within protected areas, providing regular awareness on wildlife roadkill to different road users, constructing underground tunnels in suitable areas and placement of road signs for different road users. This needs to be included in strategies and plans to mitigate the loss of biodiversity in the area. Furthermore, it is critical to proceed with roadkill monitoring in the area to avoid further biodiversity loss.
